# A Conserved Odorant Receptor Tuned to Floral Volatiles in Three Heliothinae Species

**DOI:** 10.1371/journal.pone.0155029

**Published:** 2016-05-10

**Authors:** Song Cao, Yang Liu, Mengbo Guo, Guirong Wang

**Affiliations:** State Key Laboratory for Biology of Plant Diseases and Insect Pests, Institute of Plant Protection, Chinese Academy of Agricultural Sciences, Beijing 100193, China; Duke University, UNITED STATES

## Abstract

Odorant receptors (ORs) play an important role in insects to monitor and adapt to the external environment, such as host plant location, oviposition-site selection, mate recognition and natural enemy avoidance. In our study, we identified and characterized OR12 from three closely-related species, *Helicoverpa armigera*, *Helicoverpa assulta*, *Heliothis virescens*, sharing between 90 and 98% of their amino acids. The tissue expression pattern analysis in *H*. *armigera* showed that *HarmOR12* was strongly expressed both in male and female antennae, but not in other tissues. Functional analysis performed in the heterologous *Xenopus* expression system showed that all three OR12 were tuned to six structurally related plant volatiles. Electroantennogram recordings from male and female antennae of *H*. *armigera* closely matched the data of *in vitro* functional studies. Our results revealed that OR12 has a conserved role in Heliothinae moths and might represent a suitable target for the control of these crop pests.

## Introduction

Olfaction mediates important aspects in the life of insects, such as finding host plants, selecting oviposition sites, and avoiding natural enemies [1,2]. It has been reported that plants may emit more than 1000 different volatile compounds [3]. Plants pests, especially phytophagous insects, can detect and discriminate between these numerous chemical stimuli with a highly sensitive olfactory system and make their appropriate choices [4]. Odorant receptors (ORs) play a central role in the olfactory system of insects by finely discriminating between chemical stimuli, while soluble proteins, also present in chemosensory organs, contribute to increase the sensitivity [5,6].

ORs are classic seven transmembrane domain (7-TM) proteins located on the dendrites of olfactory receptor neurons (ORNs) within specialized sensory hairs (sensilla). In each ORN a heterodimer between a conserved OR co-receptor (Orco) and a divergent, conventional ORx forms an ion channel and mediates odorant-binding specificity [7–11]. The ORx in different insects fall into different sub-families; in moths they are usually distinguished between pheromone receptors (PRs) and non-pheromone receptors (non-PRs ORs), according to their functions [12]. PRs are uniquely or preferentially expressed in male antennae and specifically tuned to conspecific pheromone components or analogues. Non-PRs ORs are often expressed in the antennae of both sexes and detect general odorants, such as plant volatiles, thought to be essential for finding food sources and oviposition sites [12]. Many ORs have been identified so far in different insect species and the functions of PRs have been clarified in some Lepidoptera [13–17]. However, non-PRs ORs, despite being important for the choice of host-plant still remain largely uncharacterized [2,4,12,18].

The Heliothinae are a subfamily of 365 species of noctuid moths [19], which include some of the world’s most serious crop pests, such as the Old World bollworm (*Helicoverpa armigera*), the New World tobacco budworm (*Heliothis virescens*) and the oriental tobacco budworm (*Helicoverpa assulta*), which cause damage amounting to billions of dollars annually [20]. These species are also regarded as good models for studying the evolution of pheromone biosynthesis and perception.

The sex pheromone components of these three moths include a blend of three components, Z-11-hexadecenal, Z-9-hexadecenal and hexadecanal but in different ratios [21–25]. However, the host-plant ranges of the three species are significantly different. *H*. *armigera* is a euryphagous insect which feeds on over 200 different kinds of plants from 40 families [26]. *H*. *assulta* is an oligophagous insect, mainly feeding on Solanaceae, such as tobacco and hot pepper [20,27]. *H*. *virescens* is a polyphagous of more than 19 species of economic relevance and at least 80 more spontaneous plants [28]. The host diversities between these three pests are most likely related to differences in their olfactory and gustatory systems.

Previous studies have confirmed that some species of moths are attracted to flowers primarily in search of nectar, and floral odors play a significant role in the nectar foraging behavior [29]. It has also been reported that linalool, a common floral volatile, is an attractant, alone or with phenylacetaldehyde to various noctuid pests [30]. Another important floral volatile, geraniol showed significant attraction to *H*. *armigera* [31]. However, the molecular mechanisms of floral volatiles reception in nectar feeding insects, especially for noctuid moths, are little known. Using a combination of genomic sequence analysis and cDNA-library screening, 18 candidate ORs of *H*. *virescens* were first reported [32,33]. More recently, antennal transcriptome analysis has provided a large number of candidate odorant receptors in *H*. *armigera* and *H*. *assulta* [34,35], including orthologues of the 18 *H*. *virescenes* ORs. The 18 ORs except six PRs, Orco and three no-full length ORs, share more than 87% of their amino acids with orthologue genes in *H*. *armigera* and *H*. *assulta* and might be involved in detecting outside odors. Therefore, we decided to functionally characterize these genes.

We have found that HarmOR12, as well as its orthologs in the other two species, were sensitive to six volatile compounds, including important floral odorants linalool and geraniol. This gene is selectively expressed in the antennae of *H*. *armigera* without significant difference between sexes. Finally, EAG recordings revealed that all the six compounds can elicit electrophysiological responses from the antennae of male and female *H*. *armigera*. Our results suggest that OR12 might play a conserved role as floral odors sensor in Heliothinae moths and could represent a candidate target for pest control.

## Materials and Methods

### Insects rearing

The *H*. *armigera* used in all experiments were obtained from a colony maintained at the Institute of Plant Protection, Chinese Academy of Agricultural Sciences, Beijing, China. Larvae were reared on an artificial diet at 27±1°C with a photoperiod of 14:10 (L: D). The *H*. *assulta* larvae were collected from the tobacco fields with the permission of the Experiment Station of Henan University of Science and Technology in Xuchang, Henan Province, China. The insects were fed with an artificial diet at a temperature of 27 ± 1°C with a photoperiod of 16: 8 (L: D).

### RNA extraction and cDNA synthesis

Different tissues were dissected 1–3 days after eclosion, immediately frozen in liquid nitrogen and stored at –70°C until extraction. Total RNA extraction was performed using Trizol reagent and following the manufacturer’s instructions (Invitrogen, Carlsbad, CA, USA). Total RNA was dissolved in RNase-free water and its quality was verified by gel electrophoresis. RNA concentration was determined on a Nanodrop ND-1000 spectrophotometer (NanoDrop products, Wilmington, DE, USA). Before cDNA synthesis, the total RNA was digested with DNase I (Fermentas, Vilnius, Lithuania) to remove trace amounts of genomic DNA. The cDNA was synthesized by RevertAid First Strand cDNA Synthesis Kit (Fermentas, Vilnius, Lithuania). The cDNA samples thus obtained from the antennae of the three moths were used as templates to clone the OR12 genes. The cDNAs of eight different tissues of *H*. *armigera* were used for semi-quantitative reverse transcription PCR (RT-PCR).

### Cloning of OR12 genes from *H*. *armigera*, *H*. *assulta* and *H*. *virescens*

The sequence of *HvirOR12* is available in Genbank (GenBank accession number: AJ748327). The sequences of *HarmOR12* and *HassOR12* were identified by antennal transcriptomic analysis in our previous work [34,35]. Specific primers were designed to clone the full-length ORFs of the three OR12 genes (S1 Table). The full-length coding sequences of OR12 genes were amplified by PCR from total antennal cDNA using primeSTAR HS DNA polymerase (Takara, Dalian, China). PCR reactions of 25 μL contained 0.25 μL primeSTAR HS DNA polymerase, 5 μL 5×PrimerSTAR Buffer, 2 μL dNTP mixture (2.5 mM each) and 0.5 μL of each primer (10 μM). The PCR reactions were carried out using a Veriti Thermal Cycler (Applied Biosystems, Carlsbad, CA, USA) under the following conditions: 94°C for 5 min; 35 cycles of 94°C for 30 s, 55°C for 45 s, 72°C for 1.5 min; 72°C for 10 min. PCR products were run on a 1.0% agarose gel, and sequences were verified after ligation into the cloning vector pEASY-Blunt (TransGen Biotech, Beijing, China).

### Sequence analysis and phylogenetic analysis

The amino acid sequences of HarmOR12, HassOR12 and HvirOR12 were aligned using DNAMAN software (Version 8, Lynnon Biosoft, Quebe, Canada). A phylogenetic tree was built on the amino sequences of the 21 ORs from *H*. *armigera* [34,35], 21 ORs from *H*. *assulta* [35] and 18 ORs from *H*. *virescens* [32,33]. The unrooted tree was constructed by the neighbor-joining method, with Poisson model, using the MEGA 5.2 software (The Biodesign Institute, Center for Evolutionary Functional Genomics, Tempe, AZ, USA). Node support was assessed using a bootstrap procedure of 1000 replicates. Dendrograms were created and colour labeled with FigTree software (http://tree.bio.ed.ac.uk/software/figtree/). The transmembrane domains of HarmOR12, HassOR12 and HvirOR12 were predicted using TMHMM Server Version 2.0 (http://www.cbs.dtu.dk/services/TMHMM/).

### Semi-quantitative reverse transcription PCR

To evaluate the expression of *HarmOR12* in different tissues of male and female *H*. *armigera*, semi-quantitative reverse transcription PCR (RT-PCR) was performed using cDNA prepared from antennae (A), maxillary palps (MP), proboscises (P), heads without chemosensory appendages (H), thoraxes (T), abdomens (AB), legs (L) and genitals (G). Primers were designed using the Primer Premier 5 software (PREMIER Biosoft International, Palo Alto, CA, USA). The ribosomal protein S3 gene-HarmRPS3 was used as a control (Genbank accession number: KT962961). RT-PCR was first performed using the primers of HarmRPS3 and the amount of each cDNA template was calibrated according to the amount of the HarmRPS3 PCR products. The primer sequences are reported in S1 Table. 2×Taq Master Mix (CWBIO, Beijing, China) was used for PCR reactions. The PCR protocol was: 94°C for 5 min, 35 cycles of 94°C for 30 s, 55°C for 30 s, and 72°C for 30 s, final step at 72°C for 10 min. PCR products were analyzed on 2.0% agarose gels. The experiment was repeated three times using three independently isolated RNA samples.

### Plant volatile compounds

All the 61 plant volatile compounds in the experiment were purchased from Sigma-Aldrich, and are listed in S2 Table. In the two-electrode voltage-clamp electrophysiological recording experiments, stock solutions (1 M) were prepared in dimethyl sulphoxide (DMSO) and stored at −20°C. Before the experiments, stock solutions were diluted in 1×Ringer’s buffer (96 mM NaCl, 2 mM KCl, 5 mM MgCl_2_, 0.8 mM CaCl_2_ and 5 mM HEPES; pH 7.6 adjusted by NaOH) to working concentration of 100 μM. 1×Ringer’s buffer containing 0.1% DMSO was used as a negative control. The six volatiles used in electroantennogram recording (EAG) experiments were dissolved in hexane and diluted to working concentration of 0.5 μg/μL. All components were diluted just before the experiments.

### Receptor expression in *Xenopus* oocytes and two-electrode voltage-clamp electrophysiological recordings

Receptor expression and electrophysiological recording were performed as described in previous reports [36,37]. The full-length ORF of *HarmOR12*, *HassOR12* and *HvirOR12* were cloned into pT7Ts vector using the restriction enzymes digestion sites. cRNA was synthesized from linearized vectors with mMESSAGE mMACHINE T7 kit (Ambion, Austin, TX, USA). Mature healthy oocytes were treated with 2 mg/mL collagenase I in washing buffer (96 mM NaCl, 2 mM KCl, 5 mM MgCl_2_, and 5 mM HEPES; pH 7.6 adjusted by NaOH) for 1 h at room temperature. Oocytes were microinjected with a mixture of 27.6 ng OR12 cRNA and 27.6 ng Orco cRNA. For expression of Orco alone, 55.2 ng of each cRNA was injected. After injection, oocytes were cultured at 18°C in 1×Ringer’s buffer supplemented with 5% dialysed horse serum, 50 μg/mL tetracycline, 100 μg/mL streptomycin and 550 μg/mL sodium pyruvate. After 4−6 days of incubation, the whole-cell currents were recorded from the injected oocytes with a two-electrode voltage clamp and recorded with an OC-725C oocyte clamp (Warner Instruments, Hamden, CT, USA) at a holding potential of −80mV. Micropipettes were filled with 3M KCl. During the recording, oocytes were challenged with a panel of 61 compounds in a random order for 15 s at a flow rate of 8 mL/min. Before next stimulus, the oocytes were washed in 1×Ringer’s buffer at 10 mL/min to allow the current to return to baseline. Data acquisition and analysis were carried out with Digidata 1440A and Pclamp10.0 software (Axon Instruments Inc., Union City, CA, USA).

### Electroantennogram recording

The antenna of 2−3 days old virgin males and females was cut at the base of the flagellum and after removing the tip, it was inserted between two glass electrodes filled with 0.1 M KCl solution. The base of the antenna was connected to the reference electrode and the tip to the recording electrode. The six volatiles used in EAG assay were dissolved in hexane and diluted to 0.5 μg/μL. The molar concentration of β-Citronellol was 3.20 × 10^−3^ M, Geraniol, (−)-Linalool and Linalool were 3.24 × 10^−3^ M, trans-2-Hexenyl acetate was 3.52 × 10^−3^ M, and 3,7-Dimethyl-3-octanol was 3.16 × 10^−3^ M. A piece of filter paper (0.5 cm × 5 cm) loaded with 10 μL solution of each compound was inserted into a Pasteur pipette to deliver the stimuli. 10 μL of analytically pure solvent (hexane) was used as blank control. Antennae were stimulated with hexane and other six compounds selected from the screening with *Xenopus* oocytes in random order.

A continuous air flow of 30 mL/s was produced by a stimulus controller (CS-55, Syntech, The Netherlands) and led over the prepared antenna through a main glass tube, the outlet of which was positioned 1 cm from the antenna. The tip of a Pasteur pipette was inserted into a small hole (3 mm diameter) on the main airflow tube. Odor stimulation was controlled by a puff of purified air (0.2 s at 10 ml/s airflow) by a stimulus controller (CS-55, Syntech, The Netherlands). EAG signals from the antenna were amplified with a 10× AC/DC headstage preamplifier (Syntech, The Netherlands) and further acquired with an Intelligent Data Acquisition Controller (IDAC-4-USB; Syntech, The Netherlands). The signals were sent to a computer and recorded with a Syntech EAG-software. After subtracting blanks obtained from the same antenna, EAG responses were analyzed using the general linear model (PROC-GLM) followed by the least-significant difference (LSD) method [38–40]. Statistical significance was determined at the *α* = 0.05 level using software SAS 9.1.

## Results

### Gene cloning and sequence analysis of *HarmOR12, HassOR12* and *HvirOR12*

**Fig 1:**
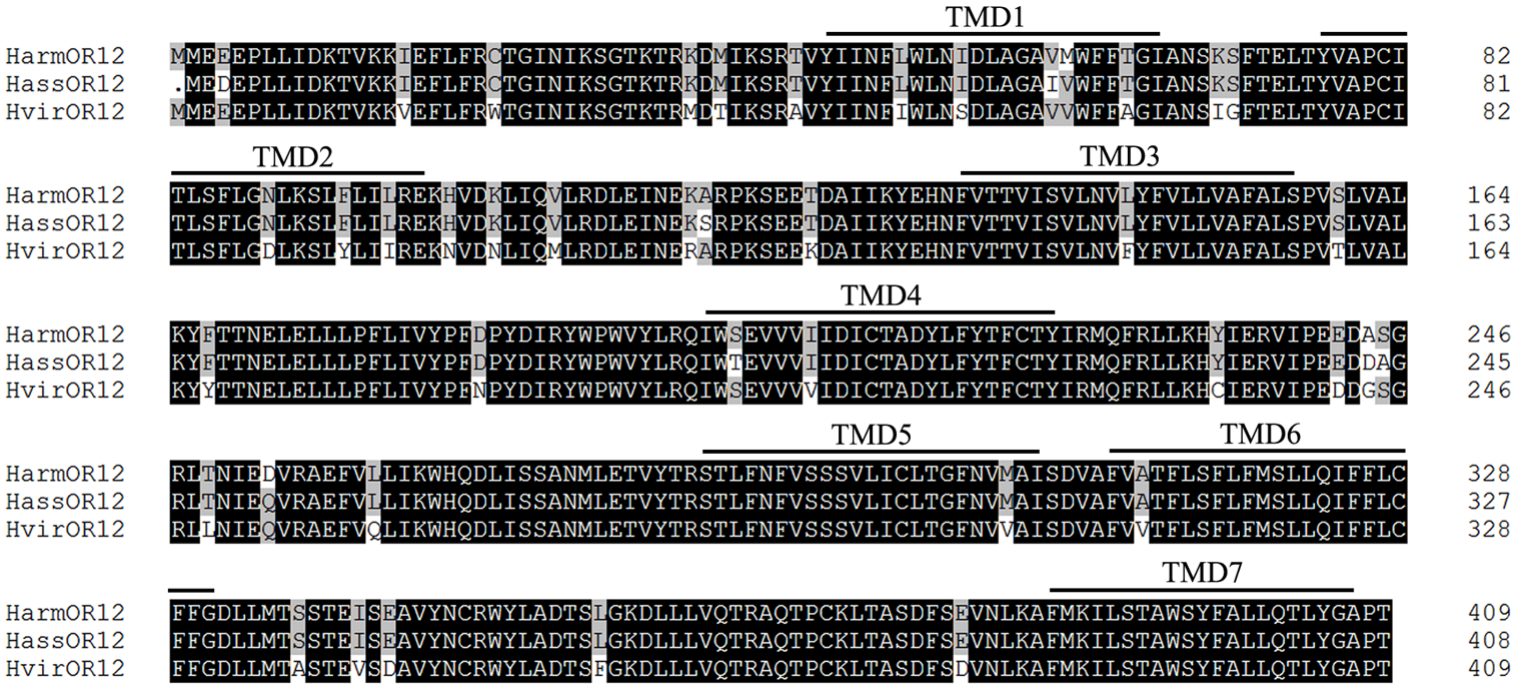
Sequence alignment of OR12 of the three species studied in this work. Percent of identical amino acids ranges between 90 and 98%. The seven transmebrane domains are marked with black lines.

**Fig 2:**
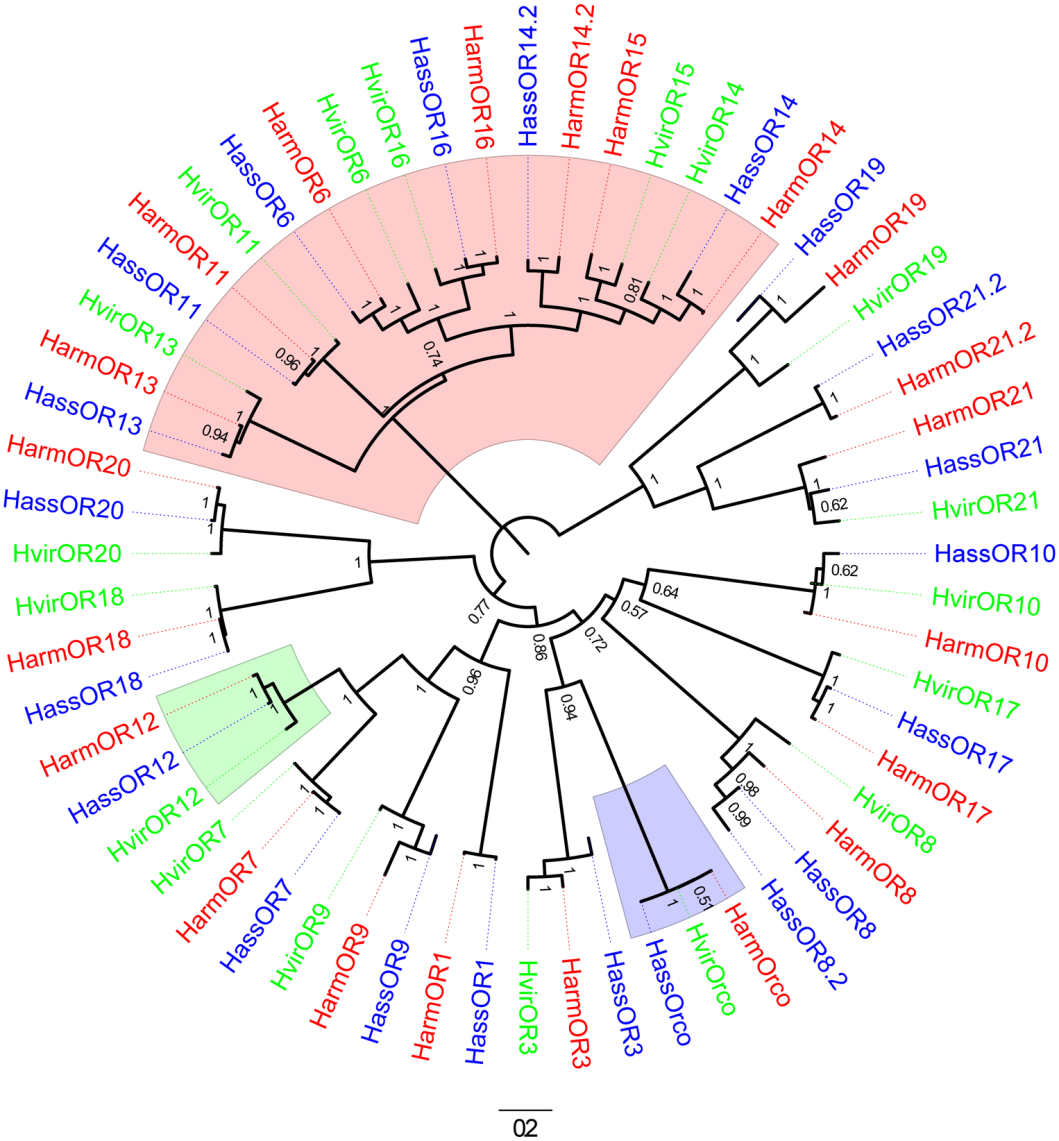
Phylogenetic tree of the ORs of the three species investigated in this work. Harm: *H*. *armigera* (red), Hass: *H*. *assulta* (blue), Hvir: *H*. *virescens* (green). The clade of PRs is highlighted in pink, that of Orco in gray and that of OR12 in light green.

The full sequences of *H*. *armigera*, *H*. *assulta* and *H*. *virescens* OR12 are 1230, 1227, 1230 bps long, encoding 409, 408, 409 amino acids, respectively. The sequences of HarmOR12 and HassOR12 have been deposited to GenBank under the accession numbers KT962962 and KT962963, respectively. As other insects ORs, these receptors present seven transmembrane domains with a predicted intracellular N-terminus and extracellular C-terminus. The three amino acid sequences are aligned in Fig 1 and are between 90% and 97.8% identical. A phylogenetic tree (Fig 2) constructed with 21 ORs from *H*. *armigera* [34,35], 21 ORs from *H*. *assulta* [35] and 18 ORs from *H*. *virescens* [32,33] clearly shows a group with the highly conserved Orco orthologues and another containing the PRs.

### Tissue expression pattern of *HarmOR12* gene

**Fig 3:**
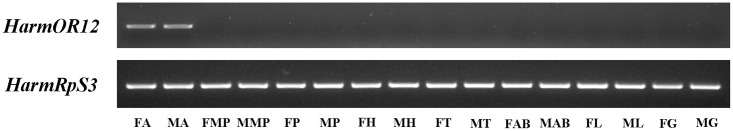
Tissue expression pattern of OR12 of *H*. *armigera*. FA: female antenna; MA: male antenna; Fmp: female maxillary palps; Mmp: male maxillary palps; FP: female proboscises; MP: male proboscises; FH: female heads; MH: male heads; FT: female thoraxes; MT: male thoraxes; Fab: female abdomens; Mab: male abdomens; FL: female legs; ML: male legs; FG: female genitalia; MG: male genitalia.

To monitor the expression pattern of OR12 in adult *H*. *armigera*, RT-PCR was carried out using cDNA templates from different tissues. A house-keeping gene encoding ribosomal protein S3 (RPS3; GenBank accession number: KT962961) was used as a control [41]. All PCR products migrated with bands of the predicted sizes and their identity was confirmed by sequencing. OR12 appears to be specifically expressed in the antennae, with no significant difference between sexes (Fig 3).

### Functional characterization of three OR12/Orco in *Xenopus* oocyte expression system

**Fig 4:**
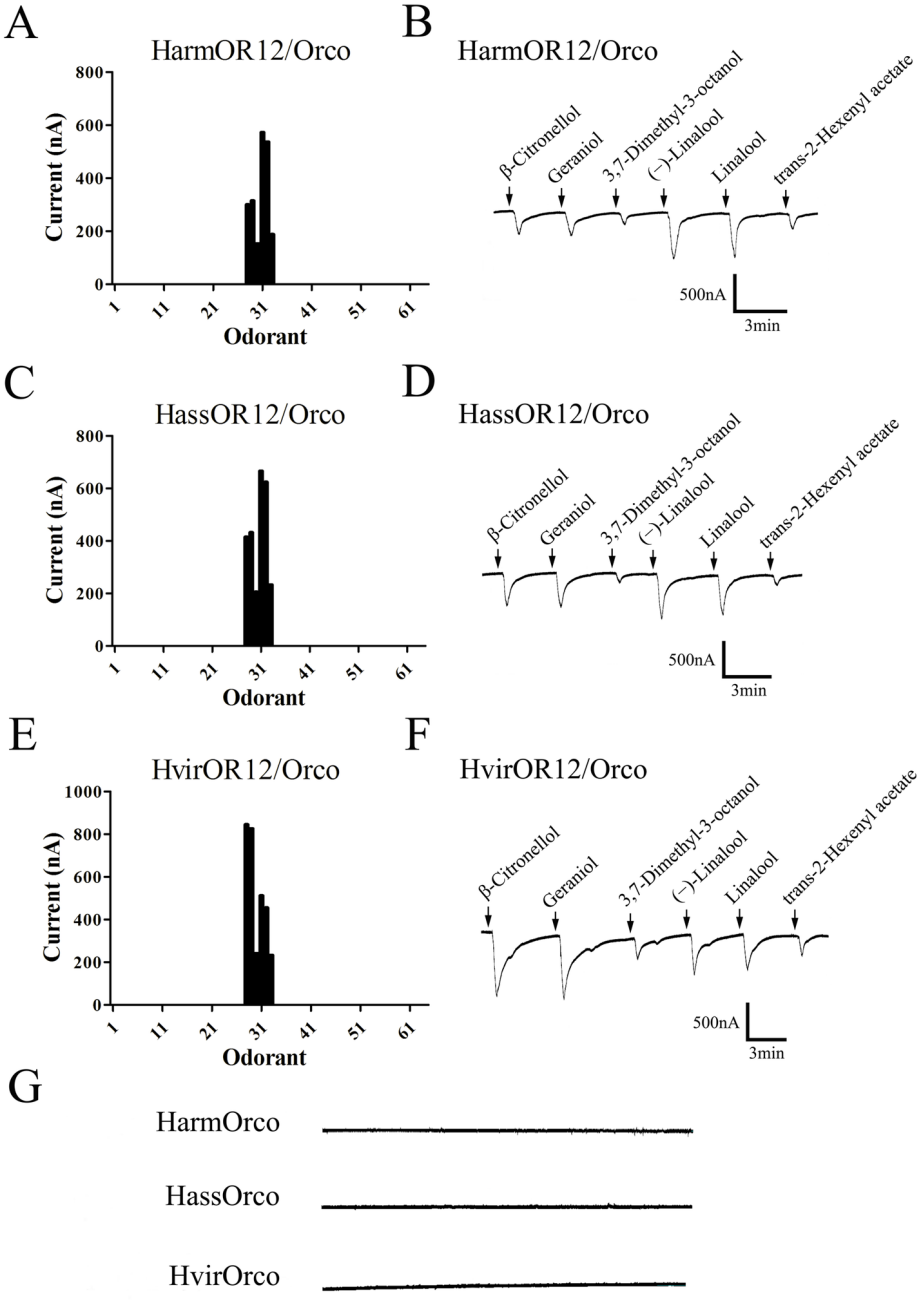
Functional characterization of three OR12/Orco in *Xenopus* oocytes. (A), (C) and (E) Tuning curves of HarmOR12/Orco, HassOR12/Orco, and HvirOR12/Orco to an odor panel comprised of 61 plant volatile compounds, arranged along the x-axis according to the strength of the response they elicit, with the strongest stimuli near the center. (B), (D) and (F) Inward current responses of HarmOR12/Orco, HassOR12/Orco and Hvir12/Orco *Xenopus* oocytes in response to 10^−4^ M solution of tested compounds, respectively. (G) Orco-injected *Xenopus* oocytes fail to respond to all the tested compounds including the six volatiles. The holding potential was −80 mV.

To identify candidate ligands, the cRNA of each OR12 was co-injected with that of Orco in *Xenopus* oocytes, and responses to odors were recorded using a two-electrode voltage clamp. This heterologous expression system has been successfully used for ORs functional studies [13,14,36,42]. Totally, we tested 61 chemicals, most of them host plant volatile compounds (listed in S2 Table) including alcohols, aldehydes, monoterpenes, sesquiterpenes and benzoates. Our results showed that the three OR12/Orco were all tuned to the same six compounds (Fig 4): β-Citronellol, Geraniol, 3,7-Dimethyl-3-octanol, (−)-Linalool, Linalool and trans-2-Hexenyl acetate (S3 Table), with no measurable response to all the other tested volatiles, even at concentrations as high as 10^−4^ M. Oocytes expressing HarmOR12/Orco and HassOR12/Orco were most sensitive to (−)-Linalool and Linalool, with responses of about 600 nA, while HvirOR12/Orco responded best to Geraniol and β-Citronellol. For all three receptors, trans-2-Hexenyl acetate and 3,7-Dimethyl-3-octanol were the weakest stimuli among the six active volatiles with responses around 200 nA.

### Electroantennogram responses

**Fig 5:**
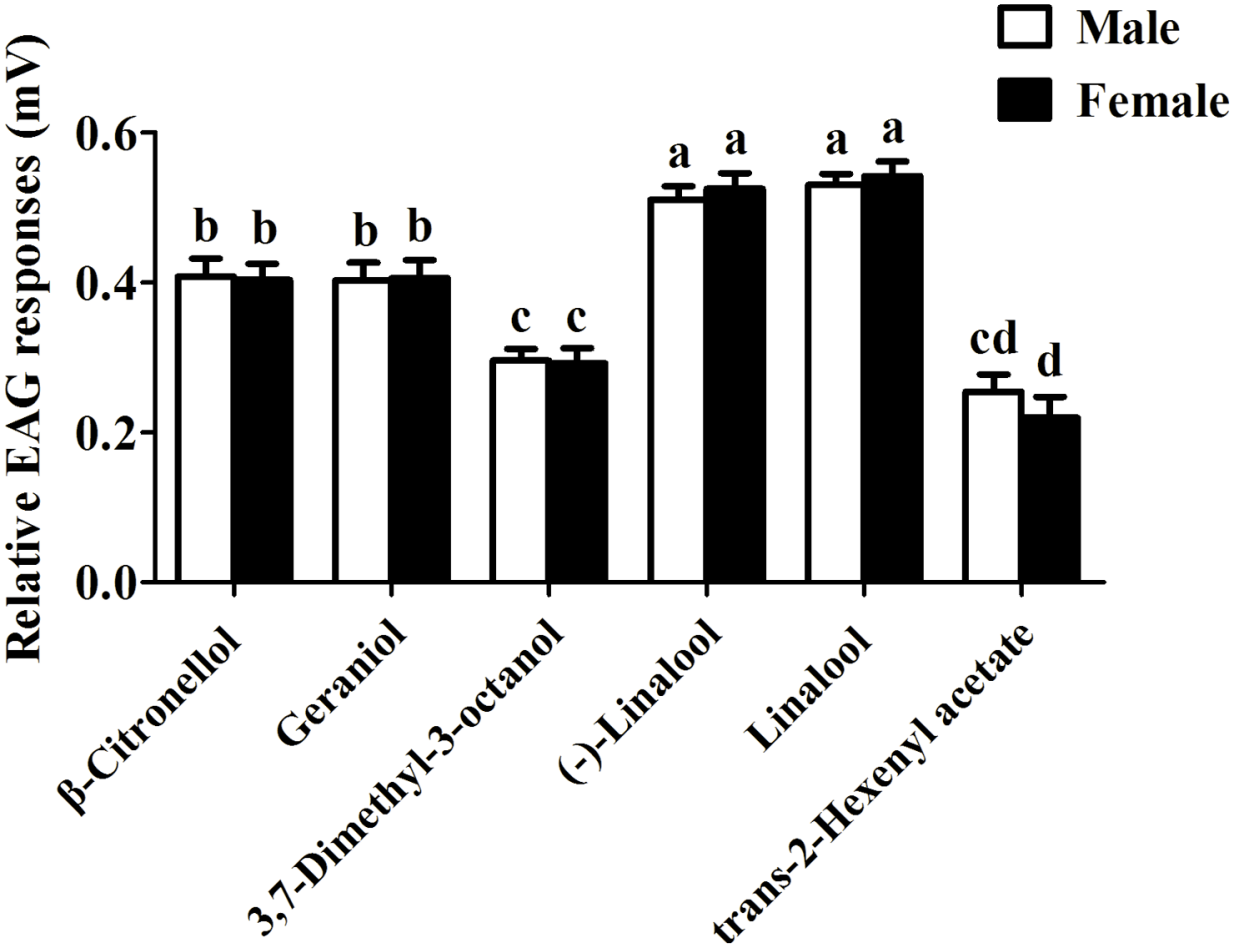
EAG responses of male and female *H*. *armigera* to six volatile compounds. The relative EAG response values marked with significant differences (*P*<0.05) among tested chemicals were analyzed by the general linear model (PROC-GLM) and followed by the least-significant difference (LSD) method. Different letters mean significant differences between components. Values are the averages of 21 biological repetitions for both males and females. Error bars indicate SEM.

We further examined EAG responses of male and female antennae of *H*. *armigera* to the six odorants found active in the *Xenopus* system and listed in S3 Table. All six volatiles elicited electrophysiological responses with no significant differences between sexes (Fig 5). In particular, (−)-Linalool and Linalool produced the strongest EAG responses, followed by β-Citronellol and Geraniol, while 3,7-Dimethyl-3-octanol and trans-2-Hexenyl acetate proved to be the weakest stimuli. Typical EAG recording obtained with negative control (Hexane) and tested volatiles are shown in S1 Fig.

## Discussion

Pheromone receptors have been well studied in several Lepidoptera during the past decade [13–17,43]. However, for non-PR ORs, represent the best part of the OR family, functional studies are very scarce. In this work, we focused on a well conserved receptor in three Heliothinae moths, OR12, which is selectively expressed in the antennae of both sexes. The fact and its selectivity for six plant volatiles support the classification of this receptor among those for general odorants. The six good stimuli are structurally similar and equally effective in eliciting electrophysiological responses from the antenna of *H*. *armigera*. The fine tuning of this receptor, which binds trans-2-Hexenyl acetate but showed no response to its isomer, cis-3-Hexenyl acetate suggests a specific role in chemical communication of the species examined.

As for the significance of the six active chemicals, it has been reported that the floral odorant geraniol is an attractant for adult *H*. *armigera*, which might use this odor cue to locate nectar sources [31]. Another floral odorant linalool is reported to be attractive or co-attractive with phenylacetaldehyde to various noctuid pests [30,31]. Another study reports that linalool is released more abundantly by plants damaged by *H*. *virescens* than those damaged by *Helicoverpa zea* [44], and suggests that this odorant could represent a chemical signal for the parasitic wasp *Cardiochiles nigriceps* to discriminate its host, *H*. *virescens*, from *H*. *zea* [44]. Linalool is also released from maize seedlings damaged by both *H*. *virescens* and *H*. *zea* [44]. We therefore speculate that this volatile may be important for *H*. *virescens* to avoid its natural enemies as well as other pests which might cause food competition. A previous report shows that the amount of geraniol released by leaves of *Persea bombycina* drastically decreased after mechanical damage or insect feeding. Another odorant used in this study, β-Citronellol is found to be abundant during larval feeding, but its concentration dropped after larvae were removed from the plants [45]. The structural similarity of geraniol and citronellol, differing only by a double bond, reasonably suggests that the two compounds activate the same receptor (S3 Table). The same specificity of OR12 in the three species suggests a conservation or reappearance of functional similarity in the related heliothine moths, independent from the evolution of euryphagy and oligophagy. Taken together, our results indicate that OR12 in the three species may play a role in location of the host plants and avoidance of natural enemies, thus representing interesting targets for environmental friendly approaches to control these economically important species.

## Supporting Information

S1 Fig Representative EAG waveforms recording in male and female *H*. *armigera* in response to olfactory stimulation with six volatiles. The sample tracings show that the amplitude of the depolarization in the baseline is nearly equal between sexes. The black arrows indicate the beginning of stimulation and the time of stimulation was 0.2 s.
(TIF)

S1 Table Primers for gene cloning, vector construction and reverse-transcription PCR. (DOCX)

S2 Table Name and CAS number of all the 61 compounds used in the functional study of HarmOR12, HassOR12 and HvirOR12. (DOCX)

S3 Table Odorants eliciting responses from the three OR12. (DOCX)
