# Supplementary figures and images for: A Conserved Odorant Receptor Tuned to Floral Volatiles in Three Heliothinae Species
Source: PLoS One. 2016 May 10;11(5):e0155029. doi: 10.1371/journal.pone.0155029 (PMC4862629; doi:10.1371/journal.pone.0155029)

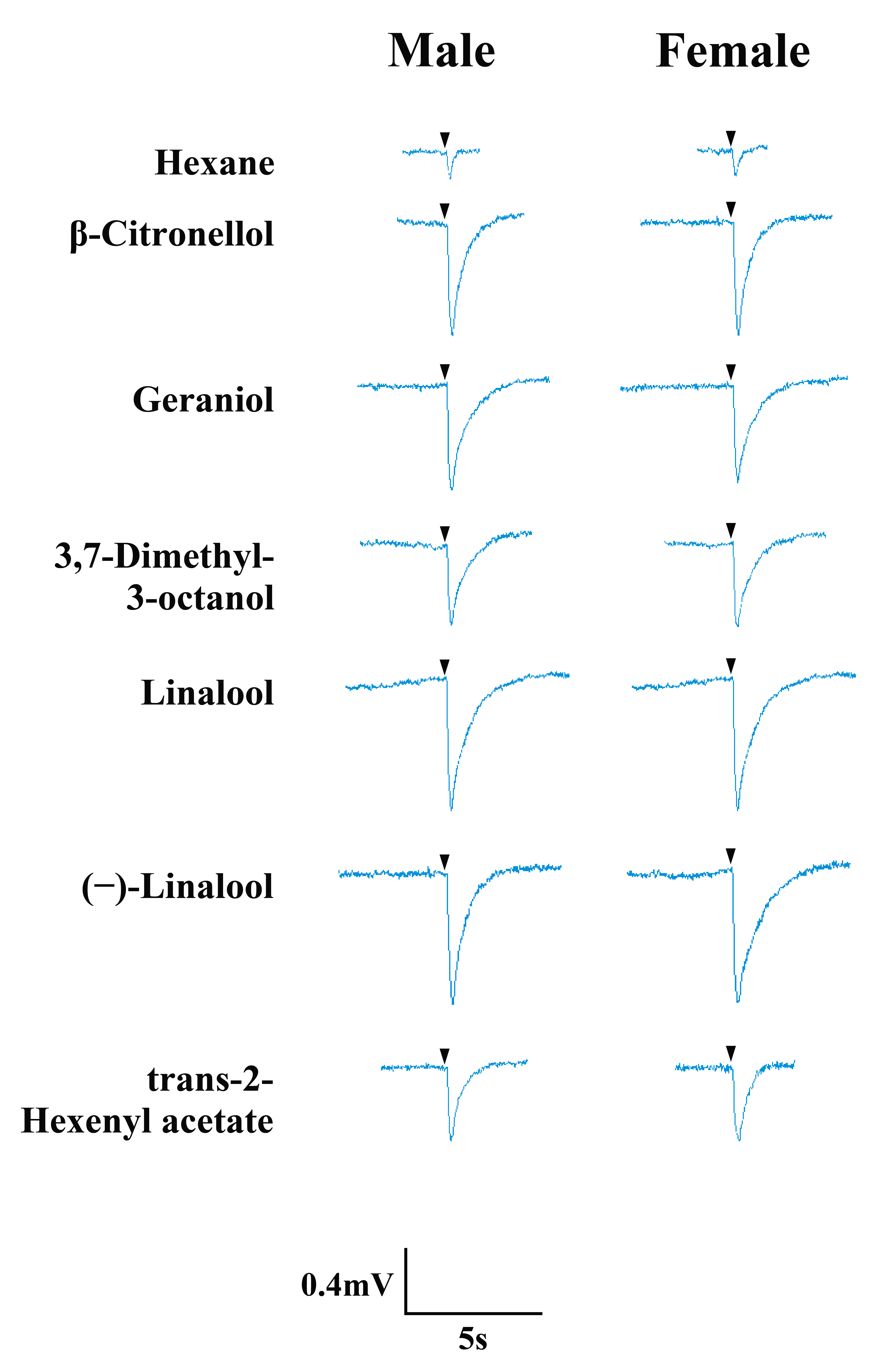

Supplement: S1 Fig — The sample tracings show that the amplitude of the depolarization in the baseline is nearly equal between sexes. The black arrows indicate the beginning of stimulation and the time of stimulation was 0.2 s. (TIF) [file pone.0155029.s001.tif]
