# Supplementary material for: A Conserved Odorant Receptor Tuned to Floral Volatiles in Three Heliothinae Species
Source: PLoS One. 2016 May 10;11(5):e0155029. doi: 10.1371/journal.pone.0155029 (PMC4862629; doi:10.1371/journal.pone.0155029)
Supplement: S1 Table — (DOCX) [file pone.0155029.s002.docx]

**S1 Table. The primers used in this study.**

| Primer name | Primer sequence (5’-3’) |
| --- | --- |
| **Gene cloning** |  |
| HarmOR12F | ATGATGGAGGAGGAACCACTG |
| HarmOR12R | TTAAGTTGGTGCTCCATAGAGG |
| HassOR12F | ATGGAGGACGAACCACTACTTATC |
| HassOR12R | ATTTTATATTTATTCCGGTGCATC |
| HvirOR12F | ATGATGGAGGAGGAACCACTG |
| HvirOR12R | GATTTTATATTTATTCCGGTCCATC |
| **Expression vector construction** | |
| HarmOR12F-E | TCAAGATCT*GCCACC*ATGATGGAGGAGGAACCACT(BglII) |
| HarmOR12R-E | TCACTCGAGTTAAGTTGGTGCTCCATAGAGG(XhoI) |
| HassOR12F-E | TCAACTAGT*GCCACC*ATGGAGGACGAACCACTACTT(SpeI) |
| HassOR12R-E | TCACTCGAGTTAAGTCGGTGCTCCGTAAA(XhoI) |
| HvirOR12F-E | TCAACTAGT*GCCACC*ATGATGGAGGAGGAACCACT(SpeI) |
| HvirOR12R-E | TCAGCATGCTTAAGTTGGTGCTCCATAGAGG(SphI) |
| **RT-PCR** |  |
| HarmRPS3RTF | GTTTTCAAGGCGGAACTCAATG |
| HarmRPS3RTR | TCATGGACTTGGCACGCTG |
| HarmOR12RTF | TTATGTGGTTTTTCACTGGTATTGC |
| HarmOR12RTR | CTATGATGACCACGACTTCTGACC |
