# Supplementary material for: A Conserved Odorant Receptor Tuned to Floral Volatiles in Three Heliothinae Species
Source: PLoS One. 2016 May 10;11(5):e0155029. doi: 10.1371/journal.pone.0155029 (PMC4862629; doi:10.1371/journal.pone.0155029)
Supplement: S2 Table — (DOCX) [file pone.0155029.s003.docx]

**S2 Table. Name and CAS number of all the 61 compounds used in the functional studies of HarmOR12, HassOR12 and HvirOR12.**

| Number | Name | CAS |
| --- | --- | --- |
| 1 | 2-Phenylethanol | 60-12-8 |
| 2 | cis-3-Hexen-1-ol | 928-96-1 |
| 3 | cis-2-Hexen-1-ol | 928-94-9 |
| 4 | 1-Heptanol | 111-70-6 |
| 5 | (1S)-(−)-Verbenone | 1196-01-6 |
| 6 | 1-Hexanol | 111-27-3 |
| 7 | (S)-cis-Verbenol | 18881-04-4 |
| 8 | trans-3-Hexen-1-ol | 928-97-2 |
| 9 | (−)-Borneol | 464-45-9 |
| 10 | (+)-Borneol | 464-43-7 |
| 11 | (1R)-(−)-Myrtenol | 19894-97-4 |
| 12 | (−)-trans-Pinocarveol | 547-61-5 |
| 13 | Ethyl hexanoate | 123-66-0 |
| 14 | Methyl benzoate | 93-58-3 |
| 15 | Myrcene | 123-35-3 |
| 16 | (R)-(+)-Limonene | 5989-27-5 |
| 17 | α-Pinene | 80-56-8 |
| 18 | (−)-β-Pinene | 18172-67-3 |
| 19 | Camphene | 79-92-5 |
| 20 | α-Humulene | 6753-98-6 |
| 21 | (S)-(−)-Limonene | 5989-54-8 |
| 22 | α-Terpinene | 99-86-5 |
| 23 | (−)-trans-Caryophyllene | 87-44-5 |
| 24 | (−)-Caryophyllen**e** oxide | 1139-30-6 |
| 25 | Farnesene, mixture of isomers |  |
| 26 | trans-2-Hexen-1-al | 6728-26-3 |
| 27 | 4-Ethylbenzaldehyde | 4748-78-1 |
| 28 | β-Citronellol | 106-22-9 |
| 29 | Geraniol | 106-24-1 |
| 30 | 3,7-Dimethyl-3-octanol | 78-69-3 |
| 31 | (−)-Linalool | 126-91-0 |
| 32 | Linalool | 78-70-6 |
| 33 | trans-2-Hexenyl acetate | 2497-18-9 |
| 34 | 3-Vinylbenzaldehyde | 19955-99-8 |
| 35 | (1R)-(−)-Myrtenal | 18486-69-6 |
| 36 | Benzaldehyde | 100-52-7 |
| 37 | Heptanal | 111-71-7 |
| 38 | cis-3-Hexenyl acetate | 3681-71-8 |
| 39 | 1,4-Diethylbenzene | 105-05-5 |
| 40 | 4-Ethylacetophenone | 937-30-4 |
| 41 | Benzyl acetate | 140-11-4 |
| 42 | Tetradecane | 629-59-4 |
| 43 | Nonyl acetate | 143-13-5 |
| 44 | Hexyl acetate | 142-92-7 |
| 45 | Ocimene | 13877-91-3 |
| 46 | Tridecane | 629-50-5 |
| 47 | Methyl salicylate | 119-36-8 |
| 48 | (±)-Camphor | 76-22-2 |
| 49 | β-Ionone | 79-77-6 |
| 50 | Nerolidol | 7212-44-4 |
| 51 | 2,6-Di-tert-butylphenol | 128-39-2 |
| 52 | 2-Pentadecanone | 2345-28-0 |
| 53 | Acetophenone | 98-86-2 |
| 54 | Cumene | 98-82-8 |
| 55 | (+)-Cedrol | 77-53-2 |
| 56 | Octyl acetate | 112-14-1 |
| 57 | Ethyl butyrate | 105-54-4 |
| 58 | Salicylaldehyde | 90-02-8 |
| 59 | Methyl 2-methoxybenzoate | 606-45-1 |
| 60 | Eugenol | 97-53-0 |
| 61 | Methyl phenylacetate | 101-41-7 |
