# Supplementary material for: A Conserved Odorant Receptor Tuned to Floral Volatiles in Three Heliothinae Species
Source: PLoS One. 2016 May 10;11(5):e0155029. doi: 10.1371/journal.pone.0155029 (PMC4862629; doi:10.1371/journal.pone.0155029)
Supplement: S3 Table — (DOCX) [file pone.0155029.s004.docx]

**S3 Table. Odorants eliciting responses from the three OR12.**

| **Name** | **CAS** | **Chemical formula** | **Structural formula** |
| --- | --- | --- | --- |
| trans-2-Hexenyl acetate | 2497-18-9 | C_8_H_14_O_2_ | 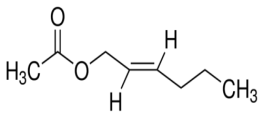 |
| β-Citronellol | 106-22-9 | C_10_H_20_O | 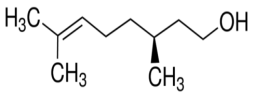 |
| Geraniol | 106-24-1 | C_10_H_18_O | 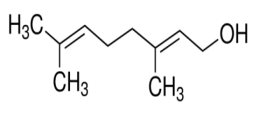 |
| 3,7-Dimethyl-3-octanol | 78-69-3 | C_10_H_22_O | 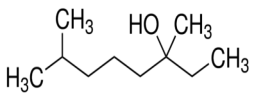 |
| Linalool | 78-70-6 | C_10_H_18_O | 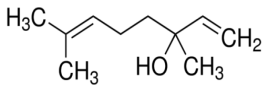 |
| (−)-Linalool | 126-91-0 | C_10_H_18_O | 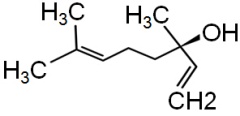 |
